# Pandemic (H1N1) 2009 Risk for Frontline Health Care Workers

**DOI:** 10.3201/eid1706.101030

**Published:** 2011-06

**Authors:** Caroline Marshall, Anne Kelso, Emma McBryde, Ian G. Barr, Damon P. Eisen, Joe Sasadeusz, Kirsty Buising, Allen C. Cheng, Paul Johnson, Michael Richards

**Affiliations:** Author affiliations: Royal Melbourne Hospital, Melbourne (C. Marshall, E. McBryde, D.P. Eisen, J. Sasadeusz, M. Richards);; University of Melbourne, Melbourne, Victoria, Australia (C. Marshall, P. Johnson);; World Health Organisation Collaborating Centre for Reference and Research on Influenza, Melbourne (A. Kelso, I.G. Barr);; St Vincent’s Hospital, Melbourne (K. Buising);; Monash University and Alfred Hospital, Melbourne (A.C. Cheng)

**Keywords:** Human influenza, health care worker–patient transmission, infection control, pandemic (H1N1) 2009, virus, Australia, research

## Abstract

To determine whether frontline health care workers (HCWs) are at greater risk for contracting pandemic (H1N1) 2009 than nonclinical staff, we conducted a study of 231 HCWs and 215 controls. Overall, 79 (17.7%) of 446 had a positive antibody titer by hemagglutination inhibition, with 46 (19.9%) of 231 HCWs and 33 (15.3%) of 215 controls positive (OR 1.37, 95% confidence interval 0.84–2.22). Of 87 participants who provided a second serum sample, 1 showed a 4-fold rise in antibody titer; of 45 patients who had a nose swab sample taken during a respiratory illness, 7 had positive results. Higher numbers of children in a participant’s family and working in an intensive care unit were risk factors for infection; increasing age, working at hospital 2, and wearing gloves were protective factors. This highly exposed group of frontline HCWs was no more likely to contract pandemic (H1N1) 2009 influenza infection than nonclinical staff, which suggests that personal protective measures were adequate in preventing transmission.

Australia was affected early in the (H1N1) 2009 influenza pandemic with 37,636 cases and 191 deaths reported. The state of Victoria was the first to observe a substantial peak in the number of persons infected (*1*). The pandemic was managed within the framework of the Australian Health Management Plan for Pandemic Influenza (*2*). Guidelines for use of personal protective equipment (PPE) were established in the Victorian Health Management Plan for Pandemic Influenza (*3*). Recommendations included use of N95 masks, gloves, protective eyewear, and long-sleeved gowns.

Influenza in health care workers (HCWs) is common, and acquisition in the workplace is well documented. An uncontrolled study found that after an influenza epidemic in Glasgow, Scotland, 120 (23.2%) of 518 HCWs seroconverted (*4*). Early in 2009, twelve HCWs with probable or possible work place acquisition of pandemic influenza were reported in the United States. None had worn full PPE (*5*).

That HCWs may be concerned about attending work during a potentially serious influenza pandemic is not surprising. During the severe acute respiratory syndrome outbreak of 2003, some HCWs reportedly stayed at home for fear of becoming infected and transmitting infection to family members. A number of surveys have found that 16%–33% of HCWs may not report to work in the event of an influenza pandemic (*6**–**9*).

HCWs need to know the transmission risks to make rational decisions about working during an influenza pandemic. Because HCWs are exposed in the community as well as the workplace, they should know about the additional risks for contracting influenza at work. This information is also imperative for pandemic workforce planning.

We sought to determine whether frontline HCWs were at greater risk for contracting pandemic (H1N1) 2009 influenza than the control group. Additionally, we sought information on factors that may have increased or decreased the risk for infection.

## Methods

We conducted a cohort study, comparing frontline HCWs with intensive patient contact (clinical) and staff with no patient contact (nonclinical). Frontline HCWs were defined as those who worked >1 shift per week and had likely exposure to patients with pandemic influenza infection. These workers included doctors, nurses, and physiotherapists, as well as others in the emergency department, intensive care unit, infectious diseases units, and respiratory and other wards where patients with suspected pandemic influenza were housed. Staff members who had no clinical contact were chosen as a convenient surrogate for a community control group. These workers included university and hospital staff in nonpatient contact areas such as the library, information technology, and administration. This study was approved by the Human Research Ethics Committees at each of the hospitals and all participants gave written informed consent. The study was conducted from August 24, 2009, through December 16, 2009.

Four tertiary referral hospitals in metropolitan Melbourne were involved: Royal Melbourne, St Vincent’s, Austin, and Alfred Hospitals. At all sites, patients with suspected or confirmed pandemic influenza infection were cared for in negative pressure isolation rooms when they were available, and in private rooms when they were not. Institutional infection control policies directed that gloves, gowns, goggles, and masks be used when caring for these patients. Use of N95 masks was initially recommended in all hospitals, although hospital 1 changed to surgical masks after June 16, 2009. Hand hygiene with an alcohol-based product and respiratory etiquette were promoted at all hospitals.

**Figure 1:**
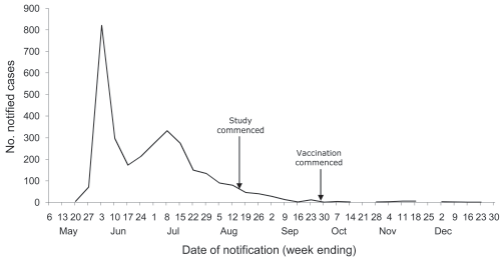
Notified cases of laboratory confirmed pandemic (H1N1) 2009, by week, Victoria, Australia, 2009. Arrows indicate dates when this study and vaccination commenced. Data provided by Victorian Department of Health, 2010.

The progression of the pandemic in Victoria is shown in Figure 1. The original research plan was to obtain 2 serum samples, 3 months apart, from all participants to test for seroconversion and also to obtain weekly nose swabs for pandemic influenza detection by using real-time PCR. By the time the study commenced, the pandemic was waning, influenza cases were decreasing in Victoria, and following the original study plan was not considered feasible.

The plan was thus modified. An initial serum sample was obtained from all participants to measure for pandemic influenza antibodies. At study entry, participants completed a Web- or paper-based questionnaire that requested information on demographic characteristics, known influenza exposures outside the workplace, and any history of fever or respiratory symptoms occurring during the pandemic but before the study. In addition, the clinical group was asked about work exposure to patients with suspected pandemic influenza and their usual use of PPE when caring for these patients. Participants were also asked about use of neuraminidase inhibitors (NIs) and specifically whether they received prophylaxis after exposure to a patient with confirmed influenza.

Participants were instructed to provide nose swab specimens for viral testing if they experienced signs and symptoms, including cough, sore throat, rhinorrhea, laryngitis, fever, myalgias, or headache. All were asked to complete a weekly questionnaire regarding symptoms, influenza exposure, and use of NIs. If a participant reported respiratory illness, a second serum sample was requested for antibody testing to document possible seroconversion.

Serum was tested for antibodies to pandemic (H1N1) 2009 influenza virus by using the hemagglutination inhibition assay with A/California/7/2009 virus and turkey red blood cells (*10*). A titer of <40 was defined as negative and >40 as positive. Nucleic acid detection was performed on nasal swabs by using reverse transcription PCR (RT-PCR) for influenza-specific and pandemic (H1N1) 2009 virus–specific sequences on swabs; kits were provided by the Centers for Disease Control and Prevention (Atlanta, GA, USA) (*11*) and an ABI-7500FAST instrument at the World Health Organization (WHO) Collaborating Centre for Reference and Research on Influenza in Melbourne.

### Statistical Analysis

On the basis of early estimates of antibody positivity to pandemic influenza virus in the community, we assumed 20% infection rates in clinical staff and 10% rates in nonclinical staff. We calculated that 438 participants were required to achieve 80% power to detect this difference using a 0.05 two-tailed significance level. The primary outcome was the presence of a positive antibody titer in the first serum sample, indicating likely pandemic influenza infection.

We performed 2 separate univariate and multivariate analyses to delineate putative risk and protective factors (1 included all participants and the other included clinical participants only) to investigate any association between health care–specific risk factors and pandemic influenza. Multivariate analysis was performed by using forward and backward stepwise logistic regression, including all variables in the model initially and a p value for removal of 0.1 and for entry of 0.2. Data were analyzed by using StataIC10 (StataCorp., College Station, TX, USA).

## Results

The study took place from August 24, 2009, through December 16, 2009, largely before release of the pandemic influenza vaccine, and no participant was vaccinated during the study. Table 1 shows the number of patients who had confirmed pandemic influenza infection (by PCR) and were treated in each of the hospitals. Characteristics of study participants are shown in Table 2.

**Table 1:** Number of patients with pandemic (H1N1) 2009 influenza virus infection at each of 4 hospitals, Australia, August 24–December 16, 2009*

Hospital no.	No. patients with confirmed pandemic (H1N1) 2009	No. inpatients	No. ICU patients	No. deaths
1	57	36	10	0
2	85	35	8	3
3	97	43	9	2
4	33	27	10	3

*ICU, intensive care unit.

**Table 2:** Characteristics of clinical and nonclinical participants at 4 hospitals at study entry (unless otherwise specified) who were infected with pandemic (H1N1) 2009, Australia, August 24–December 16, 2009*

Factor	Clinical participants, n = 231	Nonclinical participants, n = 215
Antibody titer >40	46 (19.9)	33 (15.3)
Mean age, y (range)	35.1 (19.8–56.6)	43.2 (18.5–74.1)
Female gender	157 (68.0)	153 (71.2)
Seasonal vaccination 2009	163 (70.1)	141 (65.6)
Previous seasonal vaccination	187 (80.0)	152 (70.7)
Reported confirmed pandemic (H1N1) 2009 influenza virus infection	1 (0.4)	0
Other influenza-like illness	155 (67.1)	118 (54.9)
Oseltamivir prophylaxis	13 (5.6)	1 (0.5)
Community contact with influenza	42 (18.2)	46 (21.4)
Median no. children <18 years in household (range)	0 (0–7)	0 (0–3)
Nasal swab taken during study	30 (12.9)	16 (7.4)
Mean no. hours worked per week (range)	39.2 (8–90)	37.9 (6–86)

*Values are no. (%) except as indicated.

A total of 446 HCWs participated in the study, 231 in the clinical group and 215 in the nonclinical group. Overall, 79 (17.7%) of 446 demonstrated evidence of infection on the basis of a positive antibody titer of >40, 46 (19.9%) of 231 in the clinical group, and 33 (15.3%) of 215 in the nonclinical group; the difference was not statistically significant (odds ratio [OR] = 1.37, p = 0.21, 95% confidence interval [CI] 0.84–2.22).

**Figure 2:**
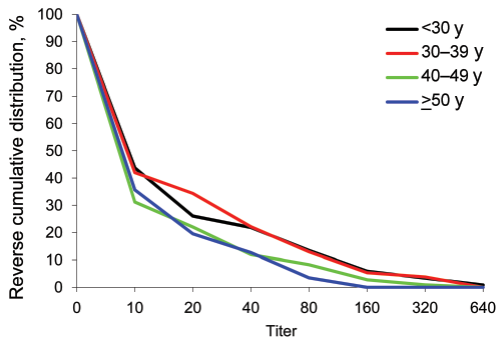
Reverse cumulative distribution of first serum antibody titer for pandemic (H1N1) 2009, by patient age, Victoria, Australia, 2009.

The median participant age was 38 years (range 18–74 years); 27% were <30 years of age, 20% were 30–39 years of age, 25% were 40–49 years of age, and 20% were >50 years of age. Figure 2 shows the reverse cumulative distribution of first serum antibody titers, according to age. We found no statistically significant difference between the curves (p = 0.11 by ordinal logistic regression).

On multivariate logistic regression, the only factor associated with a higher risk for pandemic influenza among all participants was younger age (OR 0.96, 95% CI 0.94–0.99) after adjustment for participant status (clinical vs. nonclinical), age, gender, hospital, seasonal influenza vaccination, confirmed pandemic influenza, reported respiratory illness, community contact with influenza, oseltamivir prophylaxis, number of children in the household <18 years of age, and hours worked per week. On univariate analysis, the only factors that were significantly associated with protection against infection in the clinical group were use of any mask (OR 0.16, 95% CI 0.03–0.97) and use of gloves (OR 0.09, 95% CI 0.02–0.5) for patients in droplet precautions. Adjusted odds ratios are shown in Table 3.

**Table 3:** Factors significantly associated with positive titer for pandemic (H1N1) 2009 in HCWs at 4 hospitals, Australia, August 24–December 16, 2009*

Factor	Antibody positive, n = 46	Antibody negative, n = 185	Adjusted OR (95% CI)
Mean age (range), y	33.0 (19.8–49.7)	35.6 (21.2–56.6)	0.92 (0.87–0.98)
Workplace, no. HCWs			
Emergency department	13	56	1
Infectious diseases ward	1	31	0.17 (0.02–1.48)
Intensive care unit	19	45	2.53 (1.05–6.09)
Medical ward	5	16	–
Other	6	22	–
Respiratory ward	2	15	–
Hospital no., no HCWs			
1	11	54	1
2	3	48	0.26 (0.07–0.98)
3	15	41	–
4	17	42	–
Community contact with influenza	4	38	0.25 (0.07–0.92)
Gloves for DP, no. using/no. responses	40/45	182/184	0.06 (0.01–0.46)
Median no. children <18 y in household (range)	0 (0–7)	0 (0–4)	1.83 (1.18–2.82)

*HCWs, health care workers; OR, odds ratio; CI, confidence interval; –, no result (comparator group); DP, droplet precautions. Results are adjusted for HCW status (clinical vs. nonclinical), gender, receipt of seasonal influenza vaccine, confirmed pandemic (H1N1) 2009, reported respiratory illness, oseltamivir prophylaxis, hours worked per week, work type (doctor, nurse, physiotherapist, other), work contact with influenza virus infection, mask/eye protection/gown/gloves for patients in droplet precautions, aerosol-generating procedures, and wearing an N95 mask or eye protection.

### Serology and Swab Test Results

Of the 395 participants, 140 (35%) reported a respiratory illness and 46 had nose swabs taken. Seven were positive for pandemic (H1N1) 2009 virus by PCR, 1 for subtype H3N2 influenza, and 38 were negative. One of the 46 had 2 swabs taken during different illnesses; the first was positive and the second was negative for pandemic (H1N1) 2009 virus. PCR cycle threshold values for swab specimens were from 30 to 40, indicating low viral loads. This finding may indicate that poor swabbing techniques were used, that the sample had been taken as infection was waning, or that level of infection was low (data not shown).

For 87 participants, a second serum sample was taken because of a reported respiratory illness. The average number of days between the first and second sample was 60 days (range 28 to 122 days, median 54) days. Thirty-six participants who had nose swabs performed also had a second serum sample taken. Seroconversion occurred in only 1/87 workers, with an initial titer of <10 and a subsequent titer of 40 (76 days later). This participant had a nose swab taken during a respiratory infection, which was negative for influenza virus. Seroconversion did not occur in any of the participants with a positive nose swab specimen. The mean number of days from obtaining a positive nose swab specimen to the second serum sample was 44 days (actual number: 14, 21, 27, 43, 45, 114 days). One participant with a positive nose swab sample did not have a second serum sample taken. None of the participants with a positive nose swab or seroconversion reported taking NIs in their weekly survey.

Four of the 7 participants with a positive PCR result and the 1 in whom seroconversion occurred were in the clinical group (3 doctors, 1 pharmacist, 1 nurse, 1 physiotherapist). The participant who showed seroconversion was 29 years of age; participants with a positive PCR result ranged from 24–63 years of age. Two of the participants with a positive PCR result worked on the infectious disease ward, 2 in the emergency department, and 1 in the intensive care unit; seroconversion occurred in the participant who worked in a medical ward. Five of the participants with positive PCR results and the participant in whom seroconversion occurred had received the 2009 and previous seasonal influenza vaccines. None of the participants with confirmed influenza reported taking oseltamivir for either prophylaxis or treatment.

### Weekly Questionnaires

In total, 395 participants completed 1–13 weekly questionnaires each. Eighty-nine clinical and 51 nonclinical participants reported 139 and 91 respiratory illnesses, respectively. No participant reported having laboratory-confirmed pandemic (H1N1) 2009 influenza. Six reported community contact with someone who had laboratory-confirmed infection. One reported taking oseltamivir after contact with an infected person in the workplace. This person had 2 serum samples taken 88 days apart; both had an antibody titer of <10.

## Discussion

In this study, we evaluated the risk for pandemic (H1N1) 2009 in HCWs compared with the risk for such infection in a control group, as well as the factors associated with infection. HCWs had slightly higher rates of seropositivity than nonclinical staff; however, this difference was not statistically significant. Our data are supported by results of another recent study, which found that being a HCW was not a risk factor for serologically confirmed seasonal influenza virus infection and that the risk of HCWs acquiring influenza was more strongly associated with household than workplace exposure (*12*). That study found a seroconversion rate of 11.2% in HCWs and 10.3% in non-HCWs. However, it examined only doctors and nurses, whereas our study included other types of frontline HCWs. Another study reported a seroprevalence for pandemic (H1N1) 2009 of 26.7% in HCWs, which was not significantly different from the seroprevalence of the general population (*13*). Neither of these studies examined use of PPE.

Overall, we found that 17.7% of participants had serologic evidence of pandemic (H1N1) 2009 virus infection after the peak of the outbreak. This proportion reflects the observed 16% seroprevalence in adults in Melbourne (*14*). These rates are lower, however, than the 31.7% antibody positivity found in South Australia during a prelicensure study of pandemic influenza vaccine in July 2009, which excluded subjects with confirmed or suspected pandemic (H1N1) 2009 influenza (*15*). This difference in titers may have reflected geographic differences in infection rates or differences between the populations sampled.

In the analysis of all participants, we found that older age was associated with lower rates of pandemic (H1N1) 2009 influenza infection. We did not observe higher levels of preexisting antibodies against pandemic (H1N1) 2009 influenza with increasing age, which has previously been reported. However, results of other studies examining the relationship between seroprevalence and increasing age are conflicting (*15**–**18*). Immune mechanisms other than type-specific antibodies may be providing protection for older participants. Other possibilities are that older persons have older children who may be less likely to acquire or transmit influenza or that older participants were more conscientious with respiratory etiquette and hand hygiene; attempts to measure these factors were not included in this study.

Among the HCWs we studied, working at hospital 2 conferred protection against pandemic (H1N1) 2009 virus infection. This hospital was in a geographic area with fewer cases than the others, but if this were the explanation, then a similar finding might have been expected in the nonclinical group, which was not demonstrated. Furthermore, at least as many cases of confirmed pandemic (H1N1) 2009 influenza were seen at hospital 2 as were seen at the other hospitals (Table 1). Factors such as reported compliance with PPE, were adjusted for in the multivariable analysis to reduce the effect of hospital type on influenza risk. The reason for the lower risk associated with hospital 2 has not been identified but may relate to other unmeasured factors, such as compliance with hand hygiene procedures.

Wearing gloves while caring for patients as part of droplet precautions was strongly associated with a lower risk of having had pandemic (H1N1) 2009 virus infection. Use of gloves was highly correlated with use of gowns, masks, and eye protection on logistic regression (results not shown). This finding confirms the great importance of PPE in preventing transmission of respiratory viruses in the health care setting and may explain why HCWs with definite exposure to influenza in the workplace, in addition to probable exposure in the community, do not have higher rates of infection than those with only community exposure.

The risk for pandemic (H1N1) 2009 virus infection increased with the number of children <18 years of age living in the participant’s household, which has previously been reported as a risk factor (*12*). In Victoria, the median age of persons with reported pandemic (H1N1) 2009 virus infection was 15 years, with 67% of all notified case-patients being 5–17 years of age (*1*). Miller et al. also found that children were predominantly infected (*17*). This finding, coupled with the difficulties of maintaining good respiratory etiquette in young children, is a plausible explanation for the effect of child number on infection risk.

Working in the ICU was also identified as a risk factor for pandemic influenza; patients in ICU may be severely ill, with high viral loads, and staff may be heavily exposed during multiple aerosol-generating procedures. In addition, use of PPE and hand hygiene compliance may have been lower than in other wards or patients with pandemic influenza may have been unrecognized and therefore appropriate PPE not used.

Exposure of HCWs to suspected or proven pandemic influenza in the community was protective against having a positive antibody test result. This finding is counterintuitive and difficult to explain. One hypothesis is that HCWs who knew that they had had community exposure may have been more attentive to hand hygiene and other infection control precautions while at work or were more likely to enact social distancing.

We found only 1 instance of seroconversion among the 87 participants (including the 6 with PCR-confirmed infection), each of whom had 2 serum samples taken for antibody measurement. Miller et al. reported that 89.1% of participants with pandemic (H1N1) 2009 had an antibody titer of >32 three weeks after infection, although a baseline serum sample was not taken; therefore, seroconversion could not be demonstrated (*17*). None of the participants with positive PCR results reported taking NIs, and all had serum samples taken >2 weeks after the positive nose swab specimen, allowing sufficient time for seroconversion. Our results are likely to be true positives, as all swabs were only taken when patients were symptomatic. Previously, virus isolation has been the gold standard for influenza detection but RT-PCR is now considered to be more sensitive and specific. A previous study by some of the current authors has shown that seroconversion occurs in 80%–90% of serum samples if they are tested a sufficient time after infection (confirmed by RT-PCR) (*19*). Nasal swabs are a relatively peripheral type of sample (*20*). If viral load is low in the nose, the sample may be insufficient as an antigenic stimulus to induce a detectable level of seroconversion in the serum. This may be the explanation for the lack of seroconversion seen in some PCR-positive cases in this study.

Because the number of pandemic (H1N1) 2009 cases in Victoria was low by the time this study commenced, we used a single antibody measurement for diagnosis in most patients. This is not ideal, because some participants may have had preexisting cross-reactive antibodies, as reported by others (*15**,**16*). However, this cross-reactivity has been most commonly found in older persons >65 years of age, a population which was underrepresented in our study. The explanation given for presence of cross-reactive antibodies in older persons has been past exposure to other antigenically similar viruses or a lifetime exposure to influenza A virus (*17*). Because this exposure could not have occurred in our younger study participants (median age 38 years) and serum samples were collected toward the end of the pandemic wave when many would have already been exposed, reactivity likely was specific to pandemic (H1N1) 2009. These factors support the use of a single antibody measurement for diagnosis.

This study relied on self-reported symptoms and risk factors, including use of PPE, making it subject to recall bias. This is a particular problem potentially for recall of exposures (e.g., to others with influenza or for use of PPE). However, many of the predictor variables were not subject to recall bias (e.g., clinical or nonclinical status, work place, age, gender, occupation, and number of children in the household). In addition, in order to influence the results, the 2 exposure groups would have had to exhibit differential recall. Although it could be postulated that HCWs may have perceived that they were at greater risk for exposure and may have therefore been more conscientious when filling out questionnaires, we believe that because of the large amount of public awareness of pandemic (H1N1) 2009 at that time, it is unlikely that this group would have been more conscientious than the nonclinical group.

In conclusion, we found that HCWs did not have a substantially increased risk of contracting pandemic (H1N1) 2009 in a health care setting with high availability of PPE. We conclude that use of PPE was highly protective against acquiring pandemic (H1N1) 2009 virus infection, and we therefore encourage its use, along with scrupulous hand hygiene and respiratory etiquette.
